# Role of extracellular matrix in breast cancer development: a brief update

**DOI:** 10.12688/f1000research.14133.2

**Published:** 2018-06-12

**Authors:** Manoj Kumar Jena, Jagadeesh Janjanam

**Affiliations:** 1 Department of Biotechnology, School of Bioengineering and Biosciences, Lovely Professional University , Phagwara, Punjab, 144411, India; 2 Department of Developmental Neurobiology , St. Jude Children's Research Hospital, Memphis, TN, 38105, USA

**Keywords:** Extracellular matrix, breast cancer, metastasis, matrix metalloproteinases

## Abstract

Evidence is increasing on the crucial role of the extracellular matrix (ECM) in breast cancer progression, invasion and metastasis with almost all mortality cases owing to metastasis. The epithelial-mesenchymal transition is the first signal of metastasis involving different transcription factors such as Snail, TWIST, and ZEB1. ECM remodeling is a major event promoting cancer invasion and metastasis; where matrix metalloproteinases (MMPs) such as MMP-2, -9, -11, and -14 play vital roles degrading the matrix proteins for cancer spread. The β-D mannuronic acid (MMP inhibitor) has anti-metastatic properties through inhibition of MMP-2, and -9 and could be a potential therapeutic agent. Besides the MMPs, the enzymes such as LOXL2, LOXL4, procollagen lysyl hydroxylase-2, and heparanase also regulate breast cancer progression. The important ECM proteins like integrins (b1-, b5-, and b6- integrins), ECM1 protein, and Hic-5 protein are also actively involved in breast cancer development. The stromal cells such as tumor-associated macrophages (TAMs), cancer-associated fibroblasts (CAFs), and adipocytes also contribute in tumor development through different processes. The TAMs become proangiogenic through secretion of VEGF-A and building vessel network for nourishment and invasion of the tumor mass. The latest developments of ECM involvement in breast cancer progression has been discussed in this review and this study will help researchers in designing future work on breast cancer pathogenesis and developing therapy targeted to the ECM components.

## Introduction

Breast cancer (BC) accounts for 25% of all cancer cases in women, and 12% of overall cancer cases worldwide ^1^. The extracellular matrix (ECM) plays a crucial role in BC progression, invasion, and metastasis; thus, elucidating the role of ECM will help in designing therapies targeting different ECM components. Comprehensive studies are currently going on related to the involvement of ECM in BC progression, and this review focuses on the latest developments in this regard with possible molecular targets for therapies.

The ECM (includes basement membrane (BM) and stroma) interacts with the cells mediated by ECM receptors like integrins, discoidin domain receptor, syndecans, CD44, dystoglycans, and Rhamm ^2, 3^. The BM is mainly composed of laminins (laminin- 111 is involved in milk protein synthesis and secretion), type IV collagen, entactin, and proteoglycans. The stromal cells, adipocytes, and immune cells produce many ECM proteins like type I, II, and III fibrillar collagens, fibronectin, vitronectin, elastin etc. and the stroma is highly charged and hydrated, providing tensile strength to tissues ^4^. It is observed fibrillar collagen I guides epithelial cell branching during the mammary gland development and macrophages are involved in this process of long fibre organization, required for branching morphogenesis (Rac1 acts as a modulator of collagen 1 orientation) ^5, 6^. Deregulation of the ECM dynamics is a hallmark of cancer. The ECM remodeling enzymes are deregulated changing the basic properties of ECM ^7^. There is more deposition of collagens (COL I, II, III, V, IX), and overproduction of ECM components like heparin sulphate proteoglycans and CD44 which promote growth factor signaling in cancerous cells ^8, 9^. The ECM of cancerous tissue differs from that of normal tissue in following manners: the stroma of cancerous tissue seems to be stiffer than that of normal one; the COL I fibrils in BC tissue are highly linearized, and properly oriented, whereas relaxed nonoriented fibrils are observed in normal breast tissue; many MMPs are overproduced in cancerous tissues ^7, 10– 12^. In the BC development the collagen I stiffens the ECM, thus promoting the tumor invasion and metastasis; whereas the BM prevents invasion by acting as a barrier ^3, 13, 14^.

### Epithelial-mesenchymal transition (EMT)

The EMT (process of losing epithelial characteristics and gaining mesenchymal properties) plays a significant role in the progression of tumor and metastasis, involving different transcription factors (TFs) and signals ^15– 18^. This process is characterized by loss of E-cadherin (cell-cell adhesion molecule) and cytokeratins, along with gain of N-cadherin, fibronectin, and vimentin (mesenchymal cell associated proteins) and this is termed cadherin switching (E – cadherin to N – cadherin) ^19^. The EMT is regulated by different signaling pathways such as TGF-β, notch, and wnt pathway. All these pathways converge to activate the EMT – specific TFs such as Snail (SNAI 1), slug (SNAI 2), Zeb, and Twist which differentially express in cancerous cells to promote EMT ^20– 22^. Snail is a transcriptional repressor of E-cadherin (cell-cell adhesion molecule), and E-cadherin loss is a hallmark of EMT ^2^. Snail and TWIST cooperate inducing another TF, ZEB1 ^23^ (significant inducer of EMT, invasion, and metastasis), which is triggered by extracellular hyaluronic acid (HA). Furthermore, ZEB1 induces HAS2 synthesis, promoting HA production in a positive feedback loop and its expression is correlated with ZEB1 expression in poor prognosis tumors. HAS2 also has a role in TGF-β-induced EMT ^24^. Platelets and platelet-derived TGF-β promote epithelial-mesenchymal-like transition and promote metastasis *in vivo* ^25^. Both the canonical (Smad dependent pathway) and noncanonical (Smad independent pathway) TGF-β signaling activate the TFs (Snail, Zeb, Twist, and Six1) responsible for EMT. The TGF-β induced EMT might be facilitated through enhanced expression of PARP3 (Poly ADP-Ribose Polymerase 3) protein which promotes cell motility, and chemoresistance in breast epithelial cells ^26^. The PARP3 seems to promote stemness of cancerous cells by inducing stem cell markers SOX2 and OCT4, and increasing the population of CD44 ^high^/CD24 ^low^ tumor initiating cells. The notch signaling induces the TFs (Snail, Slug, Twist, and Zeb1/Zeb2) by acting through NF-κB, promoting cytokine production and cell survival. The wnt pathway induces Snail, thus down regulates E-cadherin via β-catenin. Besides these signaling, the hypoxic microenvironment also changes the function of mitochondria leading to HIF1 stimulation and subsequently increased expression of Zeb1 required for EMT ^22^. The inflammatory factors such as TNF-α and IL-1β showed to induce plasticity in nontransformed breast epithelial cells (surrounding the transformed tumor cells) by initiating EMT through Snail and Zeb1 ^27^. Apart from this, the steroid nuclear receptors such as estrogen receptors, progesterone receptors, glucocorticoid receptors, and mineralocorticoid receptors are also observed to regulate the expression of TFs inducing EMT ^28^. A calcium - dependent phospholipid binding protein such as annexin A2 likely promotes EMT through activation of EGF/EGFR pathway. It is also observed annexin A2 directly binds to STAT3, which is a key EMT inducer up regulating the expression of TFs for EMT ^29^. Aberrant cancer metabolism promotes EMT which further aggravates metabolism (especially glucose metabolism) through Snail and Twist ^30^. One of the TF Runx1 (highly expressed in epithelium) is found to stabilize the mammary epithelial cell (MEC) phenotype thus prevents the EMT ^31^. It is observed EMT is activated by ECM stiffness, which induces the release of TWIST1 from its anchor G3BP2 and this TF enters the nucleus and transcriptionally boost the EMT process through integrin clustering and activation ^32^.

The cancer cells need to overcome anoikis (apoptosis due to loss of attachment to ECM) for metastasis event as this is a crucial barrier preventing tumor cell migration to secondary sites. Induction of anoikis occurs through lysosome – mediated down regulation of epidermal growth factor receptors (EGFRs) resulting in the termination of prosurvival signaling. It is observed the depletion of one of the kinases pre-mRNA splicing factor 4 kinase (PRP4K) promotes increased resistance to anoikis through reduced EGFR degradation (after cell detachment from ECM) with increased level of TrkB, vimentin, and ZEB1 ^33^. Anoikis is evaded in ErbB2 expressing cells by multicellular aggregation during ECM detachment. EGFRs are stabilized by this aggregation, which results in ERK/MAPK survival signaling ^34^. EGFRs could be the therapeutic targets to eliminate the ECM-detached cancer cells.

### Enzymes in ECM remodeling

Various ECM-remodeling enzymes are induced in BC promoting stem/progenitor signaling pathways and metastasis. The different pathways that are regulated by ECM during remodeling in BC development, are wnt, PI3K/AKT, ERK, JNK, Src-FAK etc ^3, 11, 35^. Major ECM proteins induced are fibrillar collagens, fibronectin, specific laminins, proteoglycans, and matricellular proteins and these could be potential drug targets for therapy ^36^. Matrix metalloproteinases (MMPs) degrade ECM proteins promoting invasion and metastasis. The MMP-11 (stromelysin-3) seems facilitating tumor development through apoptosis inhibition. However, it suppresses metastasis in animal models, exhibiting different roles in tumor progression ^37^. β-D mannuronic acid (BDM) is a MMP inhibitor, inhibiting MMP-2 and MMP-9 involved in invasion, metastasis, and angiogenesis ^38^. BDM possesses anti-metastatic activity and inhibits tumor growth by suppressing inflammatory chemokine and tumor–promoting cytokines ^39^. MMP-14 located on the cell surface, is a potential target to stop metastasis and a novel antibody-mediated MMP-14 blockade seems to limit hypoxia and metastasis in triple negative breast cancer (TNBC) models ^40^. The progression from ductal carcinoma *in situ* (DCIS) to invasive ductal carcinoma (IDC) exhibited up regulation of MMPs such as MMP-2, 11, 13, and 14 associated with invasion and ECM remodeling ^41^. The MMPs along with cross-linking enzymes LOX (Lysil oxidase) facilitate collagen maturation, regulate expression and function of soluble factor like TGF-β which ultimately reciprocates through regulation of expression of many ECM proteins and modifying enzymes including LOXs ^42^. Lox is a copper-dependent amine oxidase which initiates the intra- and intermolecular collagen crosslinking through oxidative deamination of specific lysine and hydroxylysine residues located in the telopeptide domains ^43^. This crosslinking stiffens the matrix and promotes focal adhesions (Focal adhesion kinase level is increased), integrin clustering, PI3K signaling which ultimately facilitates ErbB2 – dependent breast tumor invasion ^11^. The increased stiffness of matrix (measured by elastography) showed low response to neoadjuvant chemotherapy as compared to patients with soft breast carcinomas ^44, 45^. It is observed women with high mammographic density (MD) are more likely to develop BC as compared to women with low MD ^46^. It seems down-regulation of LOXL4 promotes BC growth and lung metastasis in mice ^47^. The LOXL2 protein catalyzes cross-linking of ECM components collagen and elastin and is involved in cancer progression and metastasis. The intracellular LOXL2 shows EMT induction and Snail-1 stabilization, and LOXL-2/Snail-1-mediated E-cadherin down-regulation promotes lung metastasis of BC without affecting ECM stiffness ^48^. Collagen is the major scaffolding protein in stroma providing tensile strength to the tissue and its metabolism is dysregulated in cancer with increased expression and deposition ^49^. The type I collagen is thought to provide barrier against tumor invasion; however enhanced collagen expression is observed with more incidence of metastasis ^50^.

The enzyme collagen prolyl hydroxylase, required for collagen synthesis, is over expressed in BC tissues with poor prognosis ^3^. Besides, the enzyme procollagen lysyl hydroxylase-2 involved in collagen synthesis, increases breast tumor stiffness, promotes metastatic tumors in lymph nodes and lungs. Matrix stiffness promotes tumor progression and invasion of ER+ type BC ^51^. The hardened ECM drives invasion and metastasis through ERK1/2 signal up-regulation and JAK2/STAT5 signal down-regulation. The enzyme heparanase cleaves heparan sulfate, promoting tumor invasion and metastasis. ER stress during chemotherapy enhances the heparanase activity ^52^. The MMTV-heparanase mice promoted growth and metastasis of breast tumor cells to lungs suggesting a role for heparanase in BC progression ^53^. Elemene (extract of C *urcuma erhizoma* plant), is an anticarcinogenic phytochemical showing effects by down-regulating heparanase expression (potential target for heparanase) ^54^. The heparin and nanoheparin derivatives show their anti-cancer activities by reducing BC cell proliferation and metastasis ^55^. Loss of ECM integrity by plasmin facilitates cancer cell spread and plasmin-induced ECM degradation may be controlled by lipoprotein-A (competitive inhibitor of plasminogen) ^56– 58^. Vitamin C seems to be very important curbing tumor growth, and metastasis as ECM integrity requires vitamin C ^58^.

The ECM proteins such as COL I, III, IV, VI, fibronectin, laminin 332, periostin, and vitronectin promote tumor progression and metastasis, whereas the proteins such as DMBT1, and SPARC suppress BC development and metastasis as reviewed by Zhu *et al*. (2014) ^3^.

### Stromal cells in BC development

Different types of stromal cells that inhabit in the tumor microenvironment (TME), are immune cells, fibroblasts, adipocytes, endothelial cells and bonemarrow derived stem cells ^59^. Tumor cells recruit tumor-associated macrophages (TAMs), which become proangiogenic by secreting VEGF-A which nourishes tumor cells and build a vessel network for their invasion. Hypoxia also induces macrophages to produce more VEGF and suppress immune response, promoting invasion ^60^. Cancer-associated fibroblasts (CAFs) are involved in tumor development, progression, inflammation, metastasis, and build resistance to cancer therapy through secretion of hormones, cytokines, growth factors, etc. and cross-talk with other stromal cells, cancer cells, and ECM. CAFs facilitate the invasion through paracrine signaling with cancer cells. The cross-talk between CAFs and cancer cells enhances IGF1 secretion by CAFs and PAI-1 (Serpine1) activity in cancer cells ^61^. These two molecules activate RhoA/ROCK signaling in cancer cells which increases cell scattering and invasion. Another study showed CAFs initially assembling an unfolded fibronectin matrix, later remodeled into a dense predominating collagen I matrix driven by MMPs ^62^. This remodeling resulted in structural and mechanical changes in the stroma, promoting proangiogenic signaling and breast tumor invasion. CAFs can be potential therapeutic targets in BC ^63^. Cancer cell proliferation and migration is induced by activated fibroblasts derived from endothelial-to-mesenchymal transformation ^64^. The cancer associated adipocytes (CAAs) have a significant role in cancer progression, ECM remodeling, phenotype changes of CAFs, and resistance to cancer therapy ^65^. They show tumor-modified phenotype with ability to modify cancer cell phenotype favoring metastasis ^66^. Comparative gene expression profiling of myoepithelial cells of cancerous (DCIS) and normal breast tissue showed up regulation of several proteases (cathepsin F, K, and L, MMP2, and PRSS19), protease inhibitors (thrombospondin2, SERPING1, cytostatin C and TIMP3), and collagens like COL1A1, COL3A1, COL6A1 in DCIS tissue ^67, 68^. 

### Various ECM proteins in BC progression

Integrins, the primary receptors of MECs for ECM, act as sensors of epithelial microenvironment. They are the transmembrane glycoproteins present as heterodimers of α- and β- subunits. Total 8 β – subunits dimerize with 18 α – subunits to form around 24 distinct integrins which specifically bind to different ECM proteins ^69^. Their altered expression seems to disorganize ECM and promotes metastasis ^70^. Increased MEC proliferation occurs due to enhanced activity of integrin signaling (β1-, β5-, and β6- integrins) by co-activating the oncogenes which augment growth factor signaling. The β1 and β3 integrins play crucial role in BC progression and metastasis, hence therapy needs targeting these two integrins at once or their downstream cytokines like FAKs (focal adhesion kinases) and SFKs (Src family kinases) for effective treatment. One of the studies revealed integrin mediated BC invasion through integrin – uPAR (urokinase/plasminogen activator urokinase receptors) signaling which leads to FRA-1 (Fos-related antigen 1) phosphorylation and invasion ^71^. The ECM protein vitronectin engagement via integrin and uPAR receptors, ends in activation of SRC and MAPK signaling which ultimately enhances FRA-1 phosphorylation. The FRA-1 (a member of AP-1 family of TFs) targets (which promote tumor cell proliferation, invasion and metastasis) include plasminogen activator, MMP-1, MMP-9, Clca2 (Chloride channel accessory2), adenosine receptor A2B, and miR221/222. Protein ECM1 is involved in angiogenesis, promoting TNBC migration and invasion ^72^. Protein Hic-5 (focal adhesion scaffold/adaptor protein) promotes mammary duct formation. Focal adhesions of cells are attached to ECM and transduce signals from ECM to cell. Hic-5 is up-regulated in CAFs of BC, involved in EMT and invadopodia (F-actin rich protrusions of cancer cells) formation facilitating invasion, migration and metastasis ^73^. The sustained directionality of tumor cells to a vessel is promoted by a chemotactic gradient of hepatocyte growth factor (HGF) produced from vessel endothelium. This directional streaming is possible by HGF/c-Met signaling pathway between endothelial cells and tumor cells; and c-Met inhibitors could be a potential target to block tumor cell streaming and metastasis ^74^.

### Tumor microenvironment (TME) as therapeutic target

Clinical trials are going on intensively at present to target the stromal cells of TME for BC therapy in combination with cancerous cell targets, reviewed recently by Bahrami *et al*. (2018) ^59^. To name a few drugs: drugs that target CAFs are chloroquine, metformin (targets lipid metabolism), anti-Met (targets glucose metabolism), celecoxib (COX-2 inhibitor), PD0332991 (cell cycle arrest), XAV 939 (β-catenin pathway inhibitor), SB431542 (TGF-β1receptor kinase inhibitor) etc. The drugs that target the immune cells are denosumab (Treg cell inhibitor), bisphosphonates (TAM inhibitor), indoximod (IDO pathway inhibitor) etc.

An anthracycline such as doxorubicin treatment of BC shows resistance to the drug mediated by ECM proteins as observed in the *in vitro* model ^75^. Hence, probably combinatorial treatment with integrin signaling inhibitors would be more effective in BC therapy. ABL kinase inhibitors like imatinib, nilotinib, and GNF-5 impede the invadopodia formation, decrease ECM degradation, and impair the matrix proteolysis-dependent invasion as observed in the mouse xenograft model ^76^. 

The cytotoxic T-cells present in the TME kill the tumor cells. However, their infiltration is retarded by various factors. The chemokines such as CXCL-9, -10, -11(production is induced by IFN-γ and chemokine gradient is established) recruit the T-cells in the TME. The tumor cells produce the extracellular galectin-3 (a lectin that binds to glycans of glycoproteins in ECM), which binds to the glycoprotein IFN-γ and prevents it from inducing secretion of above chemokines, thus impedes the cytotoxic T-cell recruitment in the TME ^77^. Immunotherapy targeting the galectin-3 would be better strategy to control tumor growth and invasion.

The TAMs (M2 phenotype) are protumoral in nature suppressing the adaptive immunity (suppresses CD8 ^+^ T cells), promoting angiogenesis, and matrix remodeling. The TAM can be a potential therapeutic target for BC therapy. Besides, the polarity switch from M2 to M1 phenotype (antitumoral function) could be a better strategy for treatment of cancer ^78, 79^.

MMP-14 was found to be a valid target to control tumor progression and metastasis in triple negative breast cancer. MMP-14 blockade by IgG3369 revealed decreased tumor neoangiogenesis and hypoxia ^80^. MMP-11 can be a crucial tumor biomarker and a potential target for immunotherapy ^81^.

High amount of hyaluronic acid (HA) present in TME seems to act as a physical barrier restricting the antibody and immune cell access to tumor cells ^82^. It was observed pericellular matrix of HA ^high^ tumor cells restricted NK cell access and antibody- dependent cell mediated cytotoxicity (ADCC). The hyaluronidase (PEGPH20) treatment showed enhanced trastuzumab-dependent ADCC and NK cell mediated tumor growth inhibition in the *in vivo* system, proving an effective adjunctive therapy for HA ^high^ tumors ^82^.

Benias *et al*. (2018) ^83^ observed presence of fluid-filled interstitial space in the submucosa of many organs (which are subjected to intermittent compression) supported by thick collagen bundles. Similar structures may also present in breast tissues facilitating the metastasis of cancer cells as opposed to dense connective tissues acting as a barrier to migrating cancer cells. 

## Conclusion

The ECM constitutes a complex of structural proteins and its reorganization is essential during cancer progression. ECM proteins provide biochemical signals to induce EMT, promote metastasis progression of cancer to advanced stage. ECM remodeling enzymes like MMPs play an essential role in these processes. The TME, platelet-derived mitogens and chemokines, granulocytes and stromal cells help cancer cells achieve intravascular transit and metastasis to target site. In addition, various ECM proteins such as integrins, collagen and fibronectin engage in cell adhesion, invasion and metastasis. All these elements of the ECM are critical for cancer progression and hence targeting ECM is a prospective approach for targeted drug discovery and cancer therapy.

## Data availability

No data is associated with this article.

## References

[1] Ferlay J Soerjomataram I Dikshit R: Cancer incidence and mortality worldwide: sources, methods and major patterns in GLOBOCAN 2012. *Int J Cancer.* 2015;136(5):E359–86. 10.1002/ijc.29210 25220842

[2] Xu R Boudreau A Bissell MJ: Tissue architecture and function: dynamic reciprocity via extra- and intra-cellular matrices. *Cancer Metastasis Rev.* 2009;28(1–2):167–176. 10.1007/s10555-008-9178-z 19160017 PMC2720096

[3] Zhu J Xiong G Trinkle C: Integrated extracellular matrix signaling in mammary gland development and breast cancer progression. *Histol Histopathol.* 2014;29(9):1083–92. 10.14670/HH-29.1083 24682974 PMC4388417

[4] Egeblad M Rasch MG Weaver VM: Dynamic interplay between the collagen scaffold and tumor evolution. *Curr Opin Cell Biol.* 2010;22(5):697–706. 10.1016/j.ceb.2010.08.015 20822891 PMC2948601

[5] Ingman WV Wyckoff J Gouon-Evans V: Macrophages promote collagen fibrillogenesis around terminal end buds of the developing mammary gland. *Dev Dyn.* 2006;235(12):3222–3229. 10.1002/dvdy.20972 17029292

[6] Brownfield DG Venugopalan G Lo A: Patterned collagen fibers orient branching mammary epithelium through distinct signaling modules. *Curr Biol.* 2013;23(8):703–709. 10.1016/j.cub.2013.03.032 23562267 PMC3705902

[7] Lu P Weaver VM Werb Z: The extracellular matrix: a dynamic niche in cancer progression. *J Cell Biol.* 2012;196(4):395–406. 10.1083/jcb.201102147 22351925 PMC3283993

[8] Huijbers IJ Iravani M Popov S: A role for fibrillar collagen deposition and the collagen internalization receptor endo180 in glioma invasion. *PLoS One.* 2010;5(3):e9808. 10.1371/journal.pone.0009808 20339555 PMC2842440

[9] Nasser NJ: Heparanase involvement in physiology and disease. *Cell Mol Life Sci.* 2008;65(11):1706–1715. 10.1007/s00018-008-7584-6 18425416 PMC11131613

[10] Lopez JI Kang I You DM: *In situ* force mapping of mammary gland transformation. *Integr Biol (Camb).* 2011;3(9):910–921. 10.1039/c1ib00043h 21842067 PMC3564969

[11] Levental KR Yu H Kass L: Matrix crosslinking forces tumor progression by enhancing integrin signaling. *Cell.* 2009;139(5):891–906. 10.1016/j.cell.2009.10.027 19931152 PMC2788004

[12] Kessenbrock K Plaks V Werb Z: Matrix metalloproteinases: regulators of the tumor microenvironment. *Cell.* 2010;141(1):52–67. 10.1016/j.cell.2010.03.015 20371345 PMC2862057

[13] Conklin MW Eickhoff JC Riching KM: Aligned collagen is a prognostic signature for survival in human breast carcinoma. *Am J Pathol.* 2011;178(3):1221–1232. 10.1016/j.ajpath.2010.11.076 21356373 PMC3070581

[14] Liotta LA Tryggvason K Garbisa S: Metastatic potential correlates with enzymatic degradation of basement membrane collagen. *Nature.* 1980;284(5751):67–68. 10.1038/284067a0 6243750

[15] Wang Y Zhou BP: Epithelial-mesenchymal transition in breast cancer progression and metastasis. *Chin J Cancer.* 2011;30(9):603–11. 10.5732/cjc.011.10226 21880181 PMC3702729

[16] De Craene B Berx G: Regulatory networks defining EMT during cancer initiation and progression. *Nat Rev Cancer.* 2013;13(2):97–110. 10.1038/nrc3447 23344542

[17] Peinado H Olmeda D Cano A: Snail, Zeb and bHLH factors in tumour progression: an alliance against the epithelial phenotype? *Nat Rev Cancer.* 2007;7(6):415–428. 10.1038/nrc2131 17508028

[18] Puisieux A Brabletz T Caramel J: Oncogenic roles of EMT-inducing transcription factors. *Nat Cell Biol.* 2014;16(6):488–494. 10.1038/ncb2976 24875735

[19] Wu Y Sarkissyan M Vadgama JV: Epithelial-Mesenchymal Transition and Breast Cancer. *J Clin Med.* 2016;5(2): pii: E13. 10.3390/jcm5020013 26821054 PMC4773769

[20] Polyak K Weinberg RA: Transitions between epithelial and mesenchymal states: acquisition of malignant and stem cell traits. *Nat Rev.* 2009;9(4):265–273. 10.1038/nrc2620 19262571

[21] Taylor MA Parvani JG Schiemann WP: The pathophysiology of epithelial-mesenchymal transition induced by transforming growth factor-beta in normal and malignant mammary epithelial cells. *J Mammary Gland Biol Neoplasia.* 2010;15(2):169–190. 10.1007/s10911-010-9181-1 20467795 PMC3721368

[22] Felipe Lima J Nofech-Mozes S Bayani J: EMT in Breast Carcinoma-A Review. *J Clin Med.* 2016;5(7): pii: E65. 10.3390/jcm5070065 27429011 PMC4961996

[23] Dave N Guaita-Esteruelas S Gutarra S: Functional cooperation between Snail1 and twist in the regulation of *ZEB1* expression during epithelial to mesenchymal transition. *J Biol Chem.* 2011;286(14):12024–32. 10.1074/jbc.M110.168625 21317430 PMC3069405

[24] Preca BT Bajdak K Mock K: A novel ZEB1/HAS2 positive feedback loop promotes EMT in breast cancer. *Oncotarget.* 2017;8(7):11530–11543. 10.18632/oncotarget.14563 28086235 PMC5355283

[25] Labelle M Begum S Hynes RO: Direct signaling between platelets and cancer cells induces an epithelial-mesenchymal-like transition and promotes metastasis. *Cancer Cell.* 2011;20(5):576–90. 10.1016/j.ccr.2011.09.009 22094253 PMC3487108

[26] Karicheva O Rodriguez-Vargas JM Wadier N: PARP3 controls TGFβ and ROS driven epithelial-to-mesenchymal transition and stemness by stimulating a TG2-Snail-E-cadherin axis. *Oncotarget.* 2016;7(39):64109–64123. 10.18632/oncotarget.11627 27579892 PMC5325429

[27] Leibovich-Rivkin T Liubomirski Y Bernstein B: Inflammatory factors of the tumor microenvironment induce plasticity in nontransformed breast epithelial cells: EMT, invasion, and collapse of normally organized breast textures. *Neoplasia.* 2013;15(12):1330–46. 10.1593/neo.131688 24403855 PMC3884524

[28] Voutsadakis IA: Epithelial-Mesenchymal Transition (EMT) and Regulation of EMT Factors by Steroid Nuclear Receptors in Breast Cancer: A Review and *in Silico* Investigation. *J Clin Med.* 2016;5(1): pii: E11. 10.3390/jcm5010011 26797644 PMC4730136

[29] Wang T Yuan J Zhang J: Anxa2 binds to STAT3 and promotes epithelial to mesenchymal transition in breast cancer cells. *Oncotarget.* 2015;6(31):30975–92. 10.18632/oncotarget.5199 26307676 PMC4741582

[30] Huang R Zong X: Aberrant cancer metabolism in epithelial-mesenchymal transition and cancer metastasis: Mechanisms in cancer progression. *Crit Rev Oncol Hematol.* 2017;115:13–22. 10.1016/j.critrevonc.2017.04.005 28602165

[31] Hong D Messier TL Tye CE: Runx1 stabilizes the mammary epithelial cell phenotype and prevents epithelial to mesenchymal transition. *Oncotarget.* 2017;8(11):17610–17627. 10.18632/oncotarget.15381 28407681 PMC5392273

[32] Wei SC Fattet L Tsai JH: Matrix stiffness drives epithelial-mesenchymal transition and tumour metastasis through a TWIST1-G3BP2 mechanotransduction pathway. *Nat Cell Biol.* 2015;17(5):678–88. 10.1038/ncb3157 25893917 PMC4452027

[33] Corkery DP Clarke LE Gebremeskel S: Loss of PRP4K drives anoikis resistance in part by dysregulation of epidermal growth factor receptor endosomal trafficking. *Oncogene.* 2018;37(2):174–184. 10.1038/onc.2017.318 28892043 PMC5770602

[34] Rayavarapu RR Heiden B Pagani N: The role of multicellular aggregation in the survival of ErbB2-positive breast cancer cells during extracellular matrix detachment. *J Biol Chem.* 2015;290(14):8722–33. 10.1074/jbc.M114.612754 25681438 PMC4423663

[35] Malanchi I Santamaria-Martinez A Susanto E: Interactions between cancer stem cells and their niche govern metastatic colonization. *Nature.* 2011;481(7379):85–89. 10.1038/nature10694 22158103

[36] Insua-Rodríguez J Oskarsson T: The extracellular matrix in breast cancer. *Adv Drug Deliv Rev.* 2016;97:41–55. 10.1016/j.addr.2015.12.017 26743193

[37] Zhang X Huang S Guo J: Insights into the distinct roles of MMP-11 in tumor biology and future therapeutics (Review). *Int J Oncol.* 2016;48(5):1783–93. 10.3892/ijo.2016.3400 26892540

[38] Mirshafiey A Khorramizadeh MR Saadat F: Chemopreventive effect of M2000, a new anti-inflammatory agent. *Med Sci Monit.* 2004;10(10):PI105–PI109. 15448610

[39] Hosseini F Hassannia H Mahdian-Shakib A: Targeting of crosstalk between tumor and tumor microenvironment by β-D mannuronic acid (M2000) in murine breast cancer model. *Cancer Med.* 2017;6(3):640–650. 10.1002/cam4.1013 28211615 PMC5345625

[40] Ling B Watt K Banerjee S: A novel immunotherapy targeting MMP-14 limits hypoxia, immune suppression and metastasis in triple-negative breast cancer models. *Oncotarget.* 2017;8(35):58372–58385. 10.18632/oncotarget.17702 28938563 PMC5601659

[41] Ma XJ Dahiya S Richardson E: Gene expression profiling of the tumor microenvironment during breast cancer progression. *Breast Cancer Res.* 2009;11(1):R7. 10.1186/bcr2222 19187537 PMC2687710

[42] Atsawasuwan P Mochida Y Katafuchi M: Lysyl oxidase binds transforming growth factor-beta and regulates its signaling via amine oxidase activity. *J Biol Chem.* 2008;283(49):34229–34240. 10.1074/jbc.M803142200 18835815 PMC2590693

[43] Yamauchi M Shiiba M: Lysine hydroxylation and cross-linking of collagen. *Methods Mol Biol.* 2008;446:95–108. 10.1007/978-1-60327-084-7_7 18373252

[44] Hayashi M Yamamoto Y Ibusuki M: Evaluation of tumor stiffness by elastography is predictive for pathologic complete response to neoadjuvant chemotherapy in patients with breast cancer. *Ann Surg Oncol.* 2012;19(9):3042–9. 10.1245/s10434-012-2343-1 22476757

[45] Evans A Armstrong S Whelehan P: Can shear-wave elastography predict response to neoadjuvant chemotherapy in women with invasive breast cancer? *Br J Cancer.* 2013;109(11):2798–802. 10.1038/bjc.2013.660 24169359 PMC3844913

[46] Nazari SS Mukherjee P: An overview of mammographic density and its association with breast cancer. *Breast Cancer.* 2018;25(3):259–267. 10.1007/s12282-018-0857-5 29651637 PMC5906528

[47] Choi SK Kim HS Jin T: LOXL4 knockdown enhances tumor growth and lung metastasis through collagen-dependent extracellular matrix changes in triple-negative breast cancer. *Oncotarget.* 2017;8(7):11977–11989. 10.18632/oncotarget.14450 28060764 PMC5355319

[48] Salvador F Martin A López-Menéndez C: Lysyl Oxidase-like Protein LOXL2 Promotes Lung Metastasis of Breast Cancer. *Cancer Res.* 2017;77(21):5846–5859. 10.1158/0008-5472.CAN-16-3152 28720577 PMC5656180

[49] Kolacna L Bakesova J Varga F: Biochemical and biophysical aspects of collagen nanostructure in the extracellular matrix. *Physiol Res.* 2007;56(Suppl 1):S51–S60. 17552894 10.33549/physiolres.931302

[50] Ramaswamy S Ross KN Lander ES: A molecular signature of metastasis in primary solid tumors. *Nat Genet.* 2003;33(1):49–54. 10.1038/ng1060 12469122

[51] Barcus CE O'Leary KA Brockman JL: Elevated collagen-I augments tumor progressive signals, intravasation and metastasis of prolactin-induced estrogen receptor alpha positive mammary tumor cells. *Breast Cancer Res.* 2017;19(1):9. 10.1186/s13058-017-0801-1 28103936 PMC5244528

[52] Li Y Liu H Huang YY: Suppression of endoplasmic reticulum stress-induced invasion and migration of breast cancer cells through the downregulation of heparanase. *Int J Mol Med.* 2013;31(5):1234–42. 10.3892/ijmm.2013.1292 23467544

[53] Boyango I Barash U Fux L: Targeting heparanase to the mammary epithelium enhances mammary gland development and promotes tumor growth and metastasis. *Matrix Biol.* 2018;65:91–103. 10.1016/j.matbio.2017.08.005 28916201

[54] Zhang Y Sun X Nan N: Elemene inhibits the migration and invasion of 4T1 murine breast cancer cells via heparanase. *Mol Med Rep.* 2017;16(1):794–800. 10.3892/mmr.2017.6638 28560389 PMC5482194

[55] Afratis NA Karamanou K Piperigkou Z: The role of heparins and nano-heparins as therapeutic tool in breast cancer. *Glycoconj J.* 2017;34(3):299–307. 10.1007/s10719-016-9742-7 27778131

[56] Rath M Pauling L: Plasmin-induced proteolysis and the role of apoprotein(a), lysine and synthetic analogs. *Orthomolecular Med.* 1992;7:17–23. Reference Source

[57] Choong PF Nadesapillai AP: Urokinase plasminogen activator system: a multifunctional role in tumor progression and metastasis. *Clin Orthop Relat Res.* 2003; (415 Suppl):S46–S58. 10.1097/01.blo0000093845.72468.bd 14600592

[58] Cha J Roomi MW Kalinovsky T: Lipoprotein(a) and vitamin C impair development of breast cancer tumors in Lp(a) ^+^; Gulo ^-/-^ mice. *Int J Oncol.* 2016;49(3):895–902. 10.3892/ijo.2016.3597 27573077 PMC4948959

[59] Bahrami A Hassanian SM Khazaei M: The Therapeutic Potential of Targeting Tumor Microenvironment in Breast Cancer: Rational Strategies and Recent Progress. *J Cell Biochem.* 2018;119(1):111–122. 10.1002/jcb.26183 28574616

[60] Obeid E Nanda R Fu YX: The role of tumor-associated macrophages in breast cancer progression (review). *Int J Oncol.* 2013;43(1):5–12. 10.3892/ijo.2013.1938 23673510 PMC3742164

[61] Daubriac J Han S Grahovac J: The crosstalk between breast carcinoma-associated fibroblasts and cancer cells promotes RhoA-dependent invasion *via* IGF-1 and PAI-1. *Oncotarget.* 2017;9(12):10375–10387. 10.18632/oncotarget.23735 29535813 PMC5828213

[62] Wang K Wu F Seo BR: Breast cancer cells alter the dynamics of stromal fibronectin-collagen interactions. *Matrix Biol.* 2017;60–61:86–95. 10.1016/j.matbio.2016.08.001 27503584 PMC5293676

[63] Jung YY Kim HM Koo JS: The role of cancer-associated fibroblasts in breast cancer pathobiology. *Histol Histopathol.* 2016;31(4):371–8. 10.14670/HH-11-700 26627101

[64] Mina SG Huang P Murray BT: The role of shear stress and altered tissue properties on endothelial to mesenchymal transformation and tumor-endothelial cell interaction. *Biomicrofluidics.* 2017;11(4):044104. 10.1063/1.4991738 28798857 PMC5533495

[65] Choi J Cha YJ Koo JS: Adipocyte biology in breast cancer: From silent bystander to active facilitator. *Prog Lipid Res.* 2018;69:11–20. 10.1016/j.plipres.2017.11.002 29175445

[66] Iyengar P Combs TP Shah SJ: Adipocyte-secreted factors synergistically promote mammary tumorigenesis through induction of anti-apoptotic transcriptional programs and proto-oncogene stabilization. *Oncogene.* 2003;22(41):6408–6423. 10.1038/sj.onc.1206737 14508521

[67] Allinen M Beroukhim R Cai L: Molecular characterization of the tumor microenvironment in breast cancer. *Cancer Cell.* 2004;6(1):17–32. 10.1016/j.ccr.2004.06.010 15261139

[68] Giussani M Merlino G Cappelletti V: Tumor-extracellular matrix interactions: Identification of tools associated with breast cancer progression. *Semin Cancer Biol.* 2015;35:3–10. 10.1016/j.semcancer.2015.09.012 26416466

[69] Pan B Guo J Liao Q: β1 and β3 integrins in breast, prostate and pancreatic cancer: A novel implication. *Oncol Lett.* 2018;15(4):5412–5416. 10.3892/ol.2018.8076 29556293 PMC5844128

[70] Glukhova MA Streuli CH: How integrins control breast biology. *Curr Opin Cell Biol.* 2013;25(5):633–41. 10.1016/j.ceb.2013.06.010 23886475 PMC3807876

[71] Annis MG Ouellet V Rennhack JP: Integrin-uPAR signaling leads to FRA-1 phosphorylation and enhanced breast cancer invasion. *Breast Cancer Res.* 2018;20(1):9. 10.1186/s13058-018-0936-8 29382358 PMC5791353

[72] Gómez-Contreras P Ramiro-Díaz JM Sierra A: Extracellular matrix 1 (ECM1) regulates the actin cytoskeletal architecture of aggressive breast cancer cells in part via S100A4 and Rho-family GTPases. *Clin Exp Metastasis.* 2017;34(1):37–49. 10.1007/s10585-016-9827-5 27770373 PMC5288287

[73] Goreczny GJ Ouderkirk-Pecone JL Olson EC: Hic-5 remodeling of the stromal matrix promotes breast tumor progression. *Oncogene.* 2017;36(19):2693–2703. 10.1038/onc.2016.422 27893716 PMC5541773

[74] Leung E Xue A Wang Y: Blood vessel endothelium-directed tumor cell streaming in breast tumors requires the HGF/C-Met signaling pathway. *Oncogene.* 2017;36(19):2680–2692. 10.1038/onc.2016.421 27893712 PMC5426963

[75] Lovitt CJ Shelper TB Avery VM: Doxorubicin resistance in breast cancer cells is mediated by extracellular matrix proteins. *BMC Cancer.* 2018;18(1):41. 10.1186/s12885-017-3953-6 29304770 PMC5756400

[76] Meirson T Genna A Lukic N: Targeting invadopodia-mediated breast cancer metastasis by using ABL kinase inhibitors. *Oncotarget.* 2018;9(31):22158–22183. 10.18632/oncotarget.25243 29774130 PMC5955141

[77] Gordon-Alonso M Hirsch T Wildmann C: Galectin-3 captures interferon-gamma in the tumor matrix reducing chemokine gradient production and T-cell tumor infiltration. *Nat Commun.* 2017;8(1):793. 10.1038/s41467-017-00925-6 28986561 PMC5630615

[78] Mantovani A Schioppa T Porta C: Role of tumor-associated macrophages in tumor progression and invasion. *Cancer Metastasis Rev.* 2006;25(3):315–22. 10.1007/s10555-006-9001-7 16967326

[79] Yang L Zhang Y: Tumor-associated macrophages: from basic research to clinical application. *J Hematol Oncol.* 2017;10(1):58. 10.1186/s13045-017-0430-2 28241846 PMC5329931

[80] Ling B Watt K Banerjee S: A novel immunotherapy targeting MMP-14 limits hypoxia, immune suppression and metastasis in triple-negative breast cancer models. *Oncotarget.* 2017;8(35):58372–58385. 10.18632/oncotarget.17702 28938563 PMC5601659

[81] Zhang X Huang S Guo J: Insights into the distinct roles of MMP-11 in tumor biology and future therapeutics (Review). *Int J Oncol.* 2016;48(5):1783–93. 10.3892/ijo.2016.3400 26892540

[82] Singha NC Nekoroski T Zhao C: Tumor-associated hyaluronan limits efficacy of monoclonal antibody therapy. *Mol Cancer Ther.* 2015;14(2):523–32. 10.1158/1535-7163.MCT-14-0580 25512619

[83] Benias PC Wells RG Sackey-Aboagye B: Structure and Distribution of an Unrecognized Interstitium in Human Tissues. *Sci Rep.* 2018;8(1): 4947. 10.1038/s41598-018-23062-6 29588511 PMC5869738

