# Supplementary material for: Role of extracellular matrix in breast cancer development: a brief update
Source: F1000Res. 2018 Jun 12;7:274. Originally published 2018 Mar 5. [Version 2] doi: 10.12688/f1000research.14133.2 (PMC6020719; doi:10.12688/f1000research.14133.2)
Supplement: Peer review history

# Peer review: Referee response for version 2

**DOI:** 10.5256/f1000research.16643.r34929

**Published:** 2018-06-26

**Authors:** Andrew R. Craig

**Affiliations:** 1 Cancer Biology & Genetics, Queen's Cancer Research Institute, Queen's University, Kingston, ON, Canada

The authors have improved the review sufficiently for indexing. While the review still suffers from a lack of depth in any particularly topic or target, it does provide a starting point for readers that are new to this field. The authors should be careful when discussing available inhibitors to relevant ECM or TME targets, as to whether they are research grade or clinical grade. Is their goal to inform future preclinical studies or clinical studies of these targets, or both?

 In future, the authors are encouraged to find particular subtopics or protein family that warrants a deeper dive into the literature.

I have read this submission. I believe that I have an appropriate level of expertise to confirm that it is of an acceptable scientific standard.

# Peer review: Referee response for version 2

**DOI:** 10.5256/f1000research.16643.r34930

**Published:** 2018-06-13

**Authors:** Yunus A. Luqmani

**Affiliations:** 1 Faculty of Pharmacy, Kuwait University, Safat, Kuwait

As I said previously this is a purely descriptive review which does not add much novelty to the existing literature. However, the authors have added additional details, as per referee comments, to improve the depth of the paper and it affords a useful succinct summary of the interaction between the cancer cell mass and its extracellular milieu which influences invasion and metastasis. On this basis I can recommend its indexing.

I have read this submission. I believe that I have an appropriate level of expertise to confirm that it is of an acceptable scientific standard.

# Peer review: Referee response for version 1

**DOI:** 10.5256/f1000research.15373.r32720

**Published:** 2018-05-09

**Authors:** Ren Xu

**Affiliations:** 1 Markey Cancer Center, Department of Pharmacology and Nutritional Sciences, University of Kentucky, Lexington, KY, USA

ECM is the major component of tumor microenvironment. The review briefly summarized function of ECM in breast cancer progression. Authors discussed function of MMPs and LOXs in ECM remodelling and cancer progression. The review also touched the roles of EMT and CAF in ECM remodelling.  However, none of these aspects has been covered thoroughly and discussed in detail.  The review provides some information about ECM and cancer progression, but lacks big picture of ECM function and detail information about ECM deposition and remodelling.

 Major comments:

1. ECM degradation is only part of ECM remodelling during cancer development. Increased ECM deposition is also associated with cancer progression. The authors need provide more and detail information about what and how ECM deposition contributes/represses cancer progression.
2. Function of ECM in cancer progression is very complicated. Some ECM proteins promote cancer progression, some may suppress cancer progression. It is crucial to classify it in the review.
3. EMT and CAF are both involved in ECM remodelling.  Detail information about how EMT and CAF regulate ECM remodelling need to be provided. It is also important to discuss the function of other stromal cells in ECM remodelling in cancer tissue.

I have read this submission. I believe that I have an appropriate level of expertise to state that I do not consider it to be of an acceptable scientific standard, for reasons outlined above.

# Response

**Published:** 2018-06-07

**Authors:** Manoj Jena

**Affiliations:** Lovely Professional University, India

All the queries have been answered in this version.

# Peer review: Referee response for version 1

**DOI:** 10.5256/f1000research.15373.r32907

**Published:** 2018-04-09

**Authors:** Yunus A. Luqmani

**Affiliations:** 1 Faculty of Pharmacy, Kuwait University, Safat, Kuwait

This is a very brief overview of the various complex processes that are thought to influence the migration of breast cancer cells from their primary site into and through the extracellular matrix, a pre-requisite for metastatic dissemination through the vascular system. The importance of the ECM in this process and its potential as a therapeutic target has only relatively recently been recognized, but the extent of interest has grown rapidly. Although this report does not add much novelty to the existing literature it is nevertheless a useful succinct summary of some of the major influencing factors/processes that are involved in breast cancer progression that inter-relates to the ECM. It is well written and easy to follow, particularly for those less familiar with this field.

 The title refers specifically to the role of the ECM. But EMT is a process occurring *within* the cancer cells prior to interaction with the ECM so it’s description is rather out of place here. Perhaps instead the authors may speculate as to how alterations or signals originating from the ECM might initiate EMT in some cells of the tumour mass (platelet derived-TGFβ is one factor mentioned but this is also secreted from cancer cells). This would add something new as little is known about the triggers (as opposed to the pleithora of mediators) of EMT. A short description of the differences in the tumour ECM from that around normal cells would be also be useful.

 Minor points:

- Statements that 98% of any cancer mortality and 90% of BC is due to metastasis should be removed or compelling evidence cited to support these figures. Whilst it may be true for BC, primary tumours in vital organs such as brain, lung, liver etc are likely to be as responsible for mortality, if not more, than their metastases.
- A few grammatical corrections: lys yl and build s and MEC abbreviation to be explained.

I have read this submission. I believe that I have an appropriate level of expertise to confirm that it is of an acceptable scientific standard, however I have significant reservations, as outlined above.

# Peer review: Referee response for version 1

**DOI:** 10.5256/f1000research.15373.r31504

**Published:** 2018-04-09

**Authors:** Andrew R. Craig

**Affiliations:** 1 Cancer Biology & Genetics, Queen's Cancer Research Institute, Queen's University, Kingston, ON, Canada

The review by Jena and Janjanam provides some updates on progress in our understanding of the complex interactions between breast cancer cells and surrounding extracellular matrix (ECM) during tumor progression and metastasis. The review also mentions the growing appreciation of the interplay of stromal cells such as tumor-associated macrophages and fibroblasts on this process. While I applaud the efforts to synthesize some recent findings in this review, each subtopic within the review would warrant a deeper dive into the literature and what this means for developing new therapies. The recent discovery of the interstitium as a fluid compartment, as opposed to a rigid ECM barrier, that facilitates metastasis within lymph nodes and blood vessels ^1^ would warrant mention in a future review on this topic. There is also evidence emerging that ECM can form a barrier to cytotoxic immune cell recruitment. What might this mean for therapies targeting matrix metalloproteinases or cancer-associated fibroblasts that alter the ECM? How can the altered ECM improve responses to chemotherapy or immunotherapy in breast cancer? 

 Overall, the topic is very interesting and timely, but the review needs to be more focused to achieve significant depth, and to truly guide thinking about new therapeutic strategies for metastatic breast cancer.

I have read this submission. I believe that I have an appropriate level of expertise to state that I do not consider it to be of an acceptable scientific standard, for reasons outlined above.

## References

[1] BeniasPCWellsRGSackey-AboagyeBKlavanHReidyJBuonocoreDMirandaMKornackiSWayneMCarr-LockeDLTheiseND: Structure and Distribution of an Unrecognized Interstitium in Human Tissues. *Sci Rep*.2018;8(1) : 10.1038/s41598-018-23062-6 4947 10.1038/s41598-018-23062-6 29588511PMC5869738
